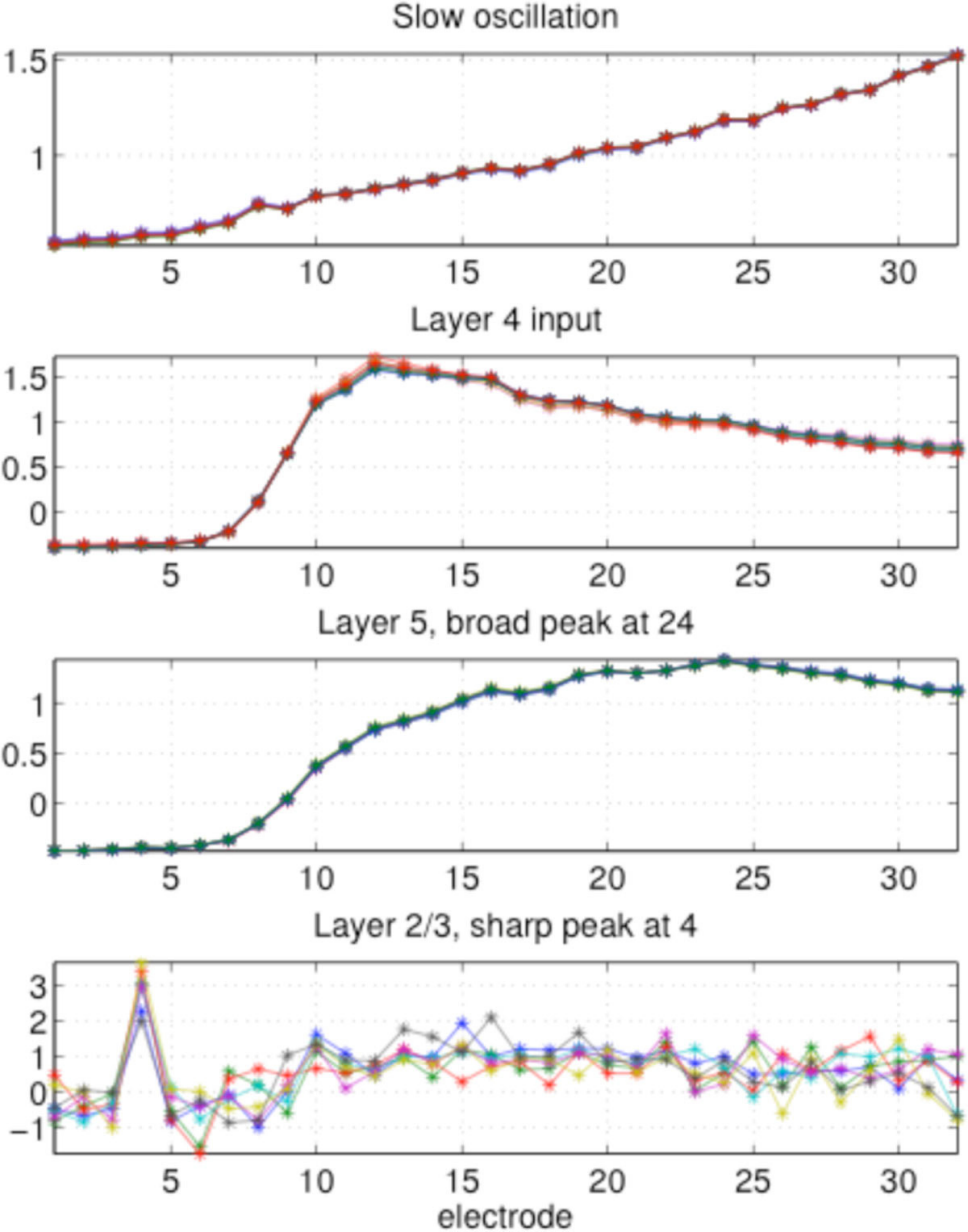# Interlaminar processing in auditory cortex before and after auditory trauma: spontaneous and evoked responses of independent sources

**DOI:** 10.1186/1471-2202-14-S1-P125

**Published:** 2013-07-08

**Authors:** Erin Munro, Shuzo Sakata, Taro Toyoizumi

**Affiliations:** 1 Lab. for Neural Computation and Adaptation, RIKEN Brain Science Institute, Wakoshi, Saitama 351-0198, Japan; 2 Strathclyde Institute of Pharmacy and Biomedical Sciences, University of Strathclyde, Glasgow, G4 0RE, UK

## 

The interaction of neural populations within the neocortex is mainly characterized by which layer they located in. For instance: thalamocortical input projects to layer 4 cells, which in turn project to layer 2/3 cell. Layer 2/3 cells then forward signals onto layer 5 cells [4]. However, it is difficult to see interactions within layers, or even which neural populations in one layer may be interacting with other layers. Very fast oscillations (VFOs, $>$80 Hz) have been associated with neocortical processing [1,3], and have distinct roles in different cortical layers [3]. Moreover, VFOs increase in temporal lobe epilepsy [2,6], which is associated with trauma [5]. In this study, we take a more detailed look at interlaminar interactions, VFOs, and the effects of trauma by applying independent component analysis (ICA) to recordings from rat auditory cortex.

**Figure 1:**